# Supplementary material for: Are carbon emissions trading and green financial instruments synergistic? -Comprehensive quantitative research based on content analysis
Source: PLoS One. 2024 Mar 7;19(3):e0298601. doi: 10.1371/journal.pone.0298601 (PMC10919601; doi:10.1371/journal.pone.0298601)
Supplement: S1 Data — (ZIP) [file pone.0298601.s001.zip › Data/地级市Z_F工作报告.zip!/╕╩╦α/╠∞╦«╩╨/└┤╫╘╣1⁄2╓┌║┼í╛┬φ┐╦╩2╛▌═°í┐_2015.docx]

各位代表:

现在,我代表市人民政府向大会报告工作,请予审议,并请各位政协委员和其他列席的同志提出意见。

2014年政府工作回顾

刚刚过去的一年,在省委、省政府和市委的坚强领导下,市政府积极应对经济下行压力加大的严峻形势,勇于担当,主动作为,趋利避害,攻坚克难,统筹推进稳增长、促改革、调结构、惠民生、防风险各项工作,经济运行稳中有升、稳中有进、稳中提质,社会事业全面进步,社会大局和谐稳定,主要经济指标增幅在全省的位次明显前移,赶超进位取得明显成效。预计生产总值503亿元,增长8.9%,其中,第一产业93亿元,增长6.1%;第二产业195.5亿元,增长10.5%;第三产业214.5亿元,增长8.5%。大口径财政收入突破100亿元,达到100.18亿元,增长15.75%,其中公共财政收入31.83亿元,增长15.31%;财政支出突破200亿元,达到201.34亿元,增长5.19%。固定资产投资540亿元,增长22%。社会消费品零售总额225亿元,增长13%。城镇居民人均可支配收入18750元,增长11%;农民人均纯收入突破5000元,增长14%。居民消费价格指数102.5%,城镇登记失业率3.2%,均低于控制目标。

——调结构,农村经济全面发展。粮食总产量123.81万吨,实现“十一连增”。果品、蔬菜、畜牧、劳务四大主导产业快速发展,新增果品面积21.02万亩、蔬菜面积5万亩、标准化规模养殖场(小区)100个,果品总产量、产值分别增长24.84%和34.6%,蔬菜总产量、产值分别增长6.38%和13.2%,畜禽饲养总量、畜牧业产值分别增长5.3%和12.5%,输转劳动力68.91万人,劳务收入97.94亿元、增长17.77%。100个农业科学发展示范点完成投资13.92亿元,天水国家农业科技园区提质扩园有序推进,市级8大农业示范园区完成投资12.1亿元,武山、甘谷蔬菜产业示范园完成核心区建设,清水核桃产业园初具规模。“甘谷鲜辣椒和辣椒面”、“甘谷干辣椒丝”、“秦安苹果”被国家工商总局认证为“地理标志证明商标”。农村土地确权登记试点有序开展,当年流转土地18.06万亩。新增产业化龙头企业76家、农民合作社567家、家庭农场173家。农村基础条件不断改善,新建农村公路2200多公里、桥梁26座1568延米,完成造林15.13万亩,新建户用沼气1254户,新修梯田34.03万亩,建成堤防工程127.94公里,治理水土流失面积360.13平方公里,解决了35.16万农村人口和5.53万农村学校师生的饮水安全问题。

——扩总量,工业经济稳中向好。预计规模以上工业总产值325亿元,增长9%;规模以上工业增加值113.16亿元,增长10%。机械制造、电工电器、电子信息、医药食品、能源化工、建筑建材六大产业集群持续壮大,增加值占全市工业增加值的75%。加快培育战略性新兴产业,20户企业列为重点培育对象,其中华天科技、长开公司、电传所公司、岐黄药业列入全省第一批重点扶持企业。实施千万元以上工业项目203个、完成投资102.5亿元,装备制造三大产业园完成投资46.2亿元,凯迪生物质能源发电项目建成运营,天水卷烟厂易地技改、张家川太极阿胶生产线二期、清水生态酿酒城技改扩建项目进展顺利,武山县干法水泥生产线、清水2×1000兆瓦火电项目有了实质进展,天水烟用包装印刷材料、通号集团铁路电缆产业升级、大型客机改货机等项目达成合作意向。天水经济技术开发区项目入园成效明显。第一批3户出城入园企业基本完成搬迁。企业自主创新能力增强,新增中国驰名商标3件、甘肃省著名商标16件、甘肃名牌产品10个,华天集团、天光半导体等企业的78件新产品(新技术)通过省级鉴定,长城电工天水电器有限责任公司获省政府质量奖。资质以内建筑业总产值94亿元、增加值59亿元,分别增长20%和11%。

——强基础,第三产业稳步发展。商贸物流基础设施日趋完善,天水商贸城、天水会展中心、秦安西部小商品城、国家级武山洛门蔬菜批发市场、清水兴盛物流园、天水城市蔬菜仓储分拣中心加快建设,天水肉类蔬菜流通追溯体系建设试点有序推进,甘谷浙江商贸城一期、张家川商业城建成运营,新建商品配送中心2个,改造提升农贸市场10个,建成便民蔬菜店50个、农家店230个。新增保险分支机构2家、证券分支机构1家,甘肃银行、交通银行升格为市级分行。银行各项存款余额898.21亿元,贷款余额475.11亿元,当年新增贷款87.08亿元。麦积山景区世行贷款项目、甘谷大像山文化主题公园、武山宁远文化产业园建设进展良好,张家川回乡风情园、秦安凤山生态公园升格为国家4A级旅游景区,麦积山温泉度假酒店建成运营,中华始祖文化园、大地湾国家考古遗址公园、麦积山文化旅游产业园前期工作积极推进。接待国内外游客1703万人次,实现旅游综合收入96.5亿元,分别增长28.02%和28.2%。

——抓改革,发展活力持续增强。坚持从政府自身改起,整合组建了市民族宗教委、市文化和旅游局、市卫生和计生委,市政府金融办由挂牌机构变为政府工作部门,市工商局、质监局实行属地管理,政府工作部门由37个精减为34个。县区政府机构改革有序推进,事业单位改革分类工作全部完成。深化行政审批制度改革,取消行政审批事项53项,承接省上下放事项39项,下放县区事项19项,市级行政审批事项年内削减63项,削减率达28.3%。新成立的市公共资源交易中心正常运行。“营改增”范围逐步扩大,减免退税1.28亿元。天水日报社和广播电视台实现经营、采编分离。出台实施扶持非公有制经济发展办法,落实注册资本认缴登记制,新增个体工商户1.48万户、增长31%,新增私营企业2235户、注册资金102.04亿元,分别增长32.6%和77%,非公有制经济占生产总值的比重达44%。重视实施关中—天水经济区发展规划,积极推进“丝绸之路经济带”宜居宜业宜游历史文化名城建设,对外交流合作不断加强,开通了天水经西安至重庆、天水经天津至大连航班,承办了第13届环青海湖国际公路自行车赛天水赛段比赛,成功举办了公祭伏羲大典暨第25届伏羲文化旅游节,并实现了两岸共祭人文始祖,伏羲文化旅游节荣获“最具国际影响力节庆”奖。有选择地参加有影响力的节会活动,招商引资成效显著,新签约招商引资项目240项,总投资510.56亿元,到位资金151.59亿元。努力扩大对外贸易,进出口总额达到3.8亿美元,增长3.3%。

——增投资,项目建设成效明显。大力实施“3341”项目工程,争取国家和省上投资项目3901项,落实资金65.48亿元。实施招商引资项目360项,到位资金312.78亿元。100个市列重大项目完成投资235.05亿元。宝天高速街亭温泉出口、武山洛门至礼县二级公路全面建成,张家川县城至恭门火车站二级公路全线贯通。我市融资建设的麦积至甘谷二级公路完成投资11亿元,占总投资的55%;引洮供水工程开工建设,落实投资6.2亿元,藉口水厂完成主体工程。中卫至贵阳天然气联络线管道天水段建成通气,750千伏输变电工程、社棠330千伏输变电工程建成投产。宝兰客运专线、天平铁路、十天高速天水段、310国道秦州至武山段升级改造、麦积全国综合养老示范基地、张家川富川水源工程和秦安小湾河水库等项目进展顺利。三年地质找矿行动初见成效,实施地质勘查项目74个,争取上级勘查资金1.56亿元。

——重建管,城乡建设步伐加快。城市总体规划修编全面启动,“一县十镇二十村”新型城镇化试点有序推进,全市城镇化率达到34.06%,提高1.6个百分点。实施城建项目284个,完成投资70.13亿元。34个市级城建重点项目当年完成投资35.38亿元。成纪新城、颖川新城、东柯新城、三阳川新区、秦州新城规划和基础设施建设有序展开,赤峪路、藉河孙家坪大桥、瀛池大桥两侧匝道和大众路、合作南北路、皇城路、光明巷等道路维修全面完工,城区5条主干道右转弯车道改造竣工使用,改造巷道130条,补修人行道3.2万平方米,新建和改造城区公厕48座,成纪大道、峡口渭河大桥、社棠渭河大桥、藉河南路西延段、赤峪路西延段、麦积新城路网等项目加快建设,双桥人行便桥建成使用。中心城区2个音乐喷泉建成使用,王家磨滨河路和山水新城景观绿化、渭河北子堤生态公园项目顺利实施,新增城市绿地45.8公顷,城区绿化覆盖率达到35.7%。秦州古城西关历史文化片区和伏羲庙、纪信祠、玉泉观、贾家公馆、后街清真寺等重点文物保护维修加快推进。农村人居环境整治逐步开展,省、市级40个示范村投入资金7.07亿元,建成整洁村360个。落实门前“三包”责任制,整治车辆乱停乱放、沿街乱搭乱建等突出问题,搬迁了藉滨花鸟鱼虫市场,“三城”联创继续推进。

——惠民生,人民生活得到改善。民生支出164.07亿元,占财政支出的81.49%。省列10项23件、市列13件民生实事全面落实。“双联”行动实施帮扶项目842项,落实资金3.7亿元,发放“双联”惠农贷款和妇女小额担保贷款2.33亿元。克服财政困难兑现了机关事业单位人员政策性增资。争取扶贫专项资金3.27亿元,实施整乡推进项目7个、整村推进项目142个,异地扶贫搬迁3852户1.97万人,减少贫困人口27万,233个村、13个乡镇实现整体脱贫。扶持就业政策全面落实,发放大学生创业贷款7800万元,帮助3500多名高校毕业生就业,新增城镇就业6.66万人。社会保险和社会救助提标扩面,城乡居民基本养老保险参保率97.6%、新农合参合率97.8%,安排城乡低保和“五保”供养资金8.47亿元,医疗救助困难群众56.7万人次、5721万元,临时救助困难群众1.8万人,发放救灾资金1443万元。甘肃救灾物资东部中心仓库、市儿童福利院、流浪未成年人救助保护中心、精神病康复医院基本完工。困难群众居住条件得到改善,争取棚户区改造贷款额度40.6亿元,新开工保障性住房和棚户区改造住房6606套,建成5257套,3934户群众入住保障房,发放廉租住房租赁补贴4492万元;实施农村危旧房改造1.28万户,受灾农村居民住房维修加固和原址恢复重建全部完成。

——促和谐,公共服务不断提升。科技创新步伐加快,取得科技创新成果107项,87项达到国内先进以上水平。新建省级工程技术研究中心2家、省级重点实验室3个。教育基础条件不断改善,实施基建项目825个,改造校舍41.26万平方米,市一中麦积校区加快建设,新建公办幼儿园33所,实现乡镇公办中心幼儿园全覆盖。文化体育事业加快发展,市文化馆、图书馆完成规划选址,市体育中心完成前期工作,新建村文化室125个、乡村舞台963个,建成体育惠民项目8个、村级农民健身项目180个。麦积山石窟“申遗”成功,清水李崖遗址被列为国家级文物保护单位,甘谷毛家坪遗址考古发掘取得重大进展。《大秦文公》、《轩辕大帝》分别获第七届中国秦腔艺术节“优秀剧目奖”、“优秀剧目特别奖”。《天水通史》出版发行。医疗卫生服务能力进一步加强,市妇幼保健院迁建、天水全科医师临床培训基地和儿童病区综合楼主体完工,建成标准化村卫生室214个,29个乡镇卫生院职工周转房项目全面竣工。落实计划生育“单独二孩”政策,人口自增率为6.08‰。统计调查、外事侨务、档案史志、防震减灾、科学储粮、人防、气象、仲裁、住房公积金等工作在服务经济社会发展中发挥了积极作用;“六五”普法、双拥共建、精神文明、妇女儿童、老龄老干、残疾人和关心下一代等工作都取得了新的成绩。深化“平安天水”建设,开展社会治安综合治理,建立综治信息平台和网上信访受理平台,完善立体化社会治安防控体系,实施社会稳定风险评估,及时妥善调处化解各类信访问题,重视加强民族宗教工作,加大食品药品安全监管力度,安全生产、交通秩序、特种设备使用专项整治有效开展,社会大局保持和谐稳定。

一年来,在市委的坚强领导下,我们坚持依法治市方略,严格依法行政,自觉接受市人大及其常委会的法律监督和工作监督、市政协的民主监督,全面执行人大决议,充分吸纳政协意见,204件人大代表意见建议、244件政协提案全部办结。扎实开展党的群众路线教育实践活动,聚焦整治“四风”顽疾,下决心治理庸懒散奢和不作为乱作为现象,工作作风不断改进,行政效能有新的提升。全面落实党风廉政建设主体责任,加大审计监督力度,强化政府督查和行政监察,纪检监察机关查处了一批违反中央“八项规定”和党纪政纪的人和事,政府系统党风廉政建设进一步加强。

需要说明的是,为适应我国经济由高速增长转向中高速增长的新常态,在省上和其他市州都将全年经济社会发展指标下调的情况下,市政府报经市委同意、提请市人大常委会审议,也将部分指标作了下调。这次下调经济指标,是主动适应新常态,积极应对新变化,统筹改革发展稳定关系,充分考虑经济指标上下对接、市州之间相互平衡、经济大环境与我市发展实际紧密结合、近期目标与长远发展有效衔接,作出的实事求是、科学合理的应对措施。

各位代表,2014年是我市经济发展赶超进位、项目建设成果丰硕、重点领域改革有效推进、民生改善成效明显的一年。这些成绩的取得,是省委、省政府正确领导、亲切关怀的结果,是市委统揽全局、科学决策的结果,是市人大及其常委会、市政协及社会各界有效监督、倾力支持的结果,是全市广大干部群众和衷共济、奋力拼搏的结果。在此,我代表市人民政府,向奋战在全市各条战线上的广大干部群众,向各民主党派、人民团体、工商联和无党派人士,向离退休老同志,向天水籍和天水工作过的在外人士,向驻市人民解放军、武警消防官兵和驻市单位,表示崇高的敬意和诚挚的感谢!

各位代表,在肯定成绩的同时,我们清醒地认识到发展中还存在不少困难和问题。主要是:经济总量不大,发展面临着保增长与调结构、转方式的双重压力,持续快速增长难度加大;招商引资引进的大项目、产业链项目少,尤其是一些县区引进的工业项目、开发性大项目少;城镇化水平不高,城乡居民人均收入增长难度较大;财政收入结构性矛盾突出,公共财政保障能力有限;一些国有商业银行存贷比低,信贷投放明显不足;去年确定的个别项目未能实施或没有达到进度要求;食品药品安全、教育、住房、医疗、社会治安等方面群众不满意的地方依然较多,整治城市脏、乱、堵任务艰巨;部分工作人员担当意识、责任意识、执行力还不强,一些人员不勤不廉的现象仍然存在。对这些问题,政府将采取有效措施,尽最大努力加以解决。

2015年奋斗目标和主要任务

2015年,是全面深化改革的关键之年,是全面推进依法治国的开局之年,也是全面完成“十二五”规划的收官之年。当前,我国经济正从高速增长转向中高速增长,经济发展方式正从规模速度型粗放增长转向质量效率型集约增长,经济结构正从增量扩能为主转向调整存量、做优增量并存的深度调整,经济发展总体向好的基本面依然没有改变。在中高速差别化发展的新常态下,我市正处于保持适当速度、继续扩张总量的追赶期,促进转型跨越、不断深化改革的攻坚期,突破瓶颈制约、彰显相对优势的潜能释放期,统筹各种资源、增进群众福祉的民生普惠期。在这关键时期,我们既面临着发展环境深刻变化的严峻挑战,也叠加了国家优先实施西部大开发、支持甘肃经济社会发展,建设丝绸之路经济带、关中—天水经济区、全省经济文化生态三个国家级创新示范区,实施六盘山片区扶贫规划,全国“两横三纵”城市化战略布局将我市列入重要节点城市等重大机遇。我们必须准确研判形势,抢抓发展机遇,凝聚发展合力,稳中求进,好中求快,以更加坚定的信心、更加主动的担当、更加有效的作为,推动全市经济社会实现新的更好更快的跨越发展。

2015年政府工作的基本思路是:认真贯彻落实中央和省委、市委一系列重大决策部署,围绕建设“丝绸之路经济带”宜居宜业宜游历史文化名城,主动适应经济发展新常态,牢牢把握发展主动权,更加注重经济发展的质量和效益,全力以赴稳增长,加大力度调结构,求实创新促改革,多策并举惠民生,科学有效防风险,统筹兼顾,突出重点,狠抓项目建设,强化工作落实,努力完成“十二五”规划确定的各项目标任务,全面加快区域性现代化大城市建设步伐。

经济社会发展的主要预期目标是:生产总值增长8.5%,规模以上工业增加值增长9%,固定资产投资增长20%,社会消费品零售总额增长12%,大口径财政收入增长10%,公共财政预算收入增长10%,城镇居民人均可支配收入增长10%,农民人均纯收入增长13%,人口自然增长率控制在7.2‰以下,居民消费价格涨幅控制在3%以内,万元生产总值综合能耗下降3.2%。

一、以项目建设为支撑,着力构建现代化大城市

(一)着力拓展城市空间。实施《甘肃省城镇体系规划》,按照“东西延伸、南北优化、相向对接、旧城改造”的总体思路,加快旧城区的整理改造,完善中心城区功能,科学谋划南北两山优化建设,加快成纪新城、颖川新城、东柯新城建设步伐,启动秦州新城建设,完善社棠工业园区城市功能,积极谋划三阳川新区、空港新城建设,全面构建区域性现代化大城市发展新格局。

(二)着力完善交通网络。充分发挥投资拉动、项目带动的作用,建成十天高速天水段、麦积至甘谷二级公路和麦积党川至两当、武山贺店至岷县2条战备公路。争取开工建设天平高速、天定高速藉口出口。加快宝兰客运专线、310国道秦州至武山段升级改造、天水西客站、麦积客运站、甘谷客运中心和清水汽车站建设。积极推进天水机场迁建、天水高铁客运枢纽站、天水至武都铁路、静宁至天水高速、陇县至甘谷高速、景泰至礼县高速公路和绕城高速改线段、清水至张家川大湾口二级公路、秦安莲花经张家川至陇县石槽沟二级公路前期工作。

(三)着力优化城市功能。加快成纪大道、赤峪路西延段、藉河南路西延段、马跑泉中心大道东延段、藉河双桥大桥、藉河山水大桥、渭河峡口一号大桥建设进度,启动实施麦积山大道、秦州至三阳川隧道、秦州大道、成纪新城路网一期、二十里铺体育中心路网、岷山厂片区路网、藉河风情线一期延伸、藉河罗峪河口大桥、藉河王家磨景观廊桥等重大项目。积极推进秦麦高速公路辅道、渭河峡口二号大桥、罗家沟大桥等项目基础工作。谋划建设城区轨道交通,完善公共自行车、公交车停靠点、公共停车场、立体停车库等设施。继续搞好商业性房产开发和保障性住房建设,重视做好市场房价调控工作。强力推动引洮供水工程,统筹实施社棠应急供水和曲溪供水项目。实施800千伏特高压直流输电工程、清水330千伏输变电站工程、天源张家川49.5兆瓦风电场和汇能甘谷古坡50兆瓦风电场项目,做好华电热电联产、清水火电、张家川火电项目前期工作。启动实施秦州热源厂、成纪新城热源厂、亚行贷款城区供热管网改造等重点供热工程。

(四)着力提升城市品位。加强历史文化名城保护,修缮保护秦州西关历史建筑,实施伏羲庙、纪信祠、木梯寺保护维修工程,做好名镇名村和传统村落保护。加快城区渭河生态环境综合整治、马跑泉公园改造、西十里藉河生态公园、山水新城滨河景观等生态环境建设项目进度,启动天水商贸城生态景观、成纪大道绿化景观、人民公园维修改造、麦积北山公园等生态景观工程建设,逐步打造市区南北两山50公里绿色长廊和藉河渭河50公里城市湿地生态长廊,增加街头花坛和公共绿地。加快“智慧城市”建设,提升城市信息化应用水平。加强宣传教育引导,提升市民文明程度。

(五)着力创新融资体系。充分利用机场迁建、城市建设、交通发展、商贸旅游等融资平台,综合运用地方政府债券和股权融资、项目融资等模式,采取特许经营、政府补助、融资代建、政府购买服务等方式,推动社会资本参与城市基础设施建设。整合优良国有资产,组建实体公司,完善多元化城市建设投融资机制。

二、以提质扩量为主线,全力促进工业转型升级

(一)深入推进工业提质扩量工程。树立集群发展理念,坚持做大增量与提升质量并重,紧盯“培育5户以上年销售收入上50亿元的产业化企业、10户以上年销售收入上10亿元的骨干企业、50户以上年销售收入上亿元的成长性企业,逐步形成年销售收入上百亿元的‘六大产业集群’,打造年销售收入上千亿元的国家级经济技术开发区”的阶段性目标,优化提升传统优势产业,积极培育战略性新兴产业,大力发展先进装备制造业,争取更多的企业进入全省战略性新兴产业扶持计划,促进特色优势产业和中小微企业集群发展。

(二)全力实施重点工业项目。加快华天电子科技园二期、长城电工电器产业园、卷烟厂易地技改、电工产品综合检测实验研究基地、武山干法水泥生产线、高盛纺织10万锭棉纺纱生产线建设,促进天森药业西北药源基地、太极集团二期阿胶生产线尽快达产达标,谋划建设工业博物馆,积极做好天水烟用包装印刷材料、蓝宝石加工、挪威小型掘进机、大型客机改货机、中航天飞扩能改造、通号集团铁路电缆升级、娃哈哈二期、艾复康生产线等重大项目的跟踪落地,工业固定资产投资增长25%以上。

(三)积极推动“一区多园”发展。加快县区工业园区基础建设,按“一区多园”共享国家级开发区优惠政策,带动县域工业发展。实施甘肃建投天水总部经济城项目,启动甘谷片区10平方公里开发区扩区增容,完善社棠工业园服务功能,发挥产业孵化园承载作用,促进入园企业投产达标,开发区工业增加值增长16%。加大协调督促力度,加快第二批、第三批出城入园企业的搬迁改造。

(四)鼓励企业加快技术创新。支持企业设立研发机构,加大科技研发投入,加快“两化”融合步伐,推广应用“西北中小企业云平台”,促进“数字企业”建设。高度重视质量发展,整合检验检测认证资源,支持天水国家智能电网输变电设备质量监督检验中心发展,推进“全国电工电器产业知名品牌示范区”建设,创建“全国质量强市示范城市”。争创省级以上企业技术中心2家、省级和国家级技术创新示范企业2家、省级以上名牌产品8件。实施商标战略,争创中国驰名商标2件、地理标志证明商标2件、甘肃省著名商标10件。

三、以现代农业为引领,推动农业增效农民增收

(一)推进“48113”现代农业计划。用工业化理念谋划农业发展,认真落实我市“48113”现代农业推进计划,逐步打造果品、蔬菜、畜牧、劳务四大百亿元主导产业,加大农业实用技术推广应用力度,推进天水国家农业科技园区提质扩园,完善市级八大农业示范园区功能,实施十个“百万工程”,培育壮大“百强龙头企业”,加快三条城乡一体化示范长廊建设。持续抓好100个农业科学发展示范点建设。

(二)做大做强特色优势产业。以果品提质、蔬菜增效、养殖扩量、劳务增收为重点,建设武山桦林苹果矮化密植示范园,实施甘谷、武山渭河川道20万亩设施蔬菜大棚改造,当年新建优质果园10万亩,新增优质蔬菜5万亩,新建标准化养殖场100个,有组织输转劳务人员70万人,劳务收入突破100亿元。加快马铃薯、中药材、花卉等特色产业发展,支持成纪、岐黄、太极等制药企业拓展种养基地,新建中药材基地3万亩。

(三)支持发展适度规模经营。引导农村土地经营权规范有序流转,通过流转土地创建50个特色农业示范点,带动农业集约化生产、规模化经营、产业化发展。支持发展农民合作社、家庭农场、经营大户等各类新型农业经营主体,当年重点培育发展龙头企业60家,新登记注册农民合作社210家、规范扶持41家,新发展家庭农场80家。

(四)持续改善农业基础条件。加强基本农田保护,加大防霜机、防雹网推广应用力度,种植全膜双垄沟播作物104万亩,力争正常年景粮食总产达到120万吨以上。加快农村沼气集中供气和“进棚入园”,实施新一轮退耕还林、“天保”二期、“三北”五期防护林、国家生态公益林等重点林业生态工程,完成各类造林20万亩,兴修梯田36万亩,新增有效灌溉面积1.2万亩、节水灌溉面积2万亩,修建堤防工程120公里,治理水土流失面积300平方公里。

(五)有效推进农村精准扶贫。坚持扶贫攻坚与“双联”行动深度融合,认真实施“1461”扶贫攻坚工程,整乡整村整流域连片开发与到村到户精准扶贫相结合,整合项目资金,加大社会帮扶,向上争取专项扶贫资金3.8亿元,实施整村推进项目130个,整乡推进项目7个,确保当年脱贫18万人,实现587个村、40个乡镇整体脱贫。

四、以扩大消费为重点,提升第三产业发展水平

(一)推动文化旅游融合发展。以华夏文明传承创新区建设“136”工程为统揽,深度挖掘利用五大历史文化资源,搭建融资平台,培育骨干企业,壮大文化产业实力,力争文化产业增加值增长31%以上。抓紧编制《天水市文化与旅游深度融合发展规划纲要》、《丝绸之路麦积山大景区建设规划》、《麦积山风景名胜区总体规划》、《大地湾国家考古遗址公园规划》和《陇东南国家中医药养生保健旅游创新区天水片区规划》。积极推进中华始祖文化园、麦积山石窟艺术研究所科研基地、秦风民俗园、黄河雕漆文化产业园、麦积山影视产业园暨游客集散中心、汉唐陶艺文化产业示范基地、佳·水岸小镇、秦州印象、西部印象、张家川回乡风情园二期、麦积百花小镇等重点项目。加快成纪新城、麦积财富阳光、甘谷冀城、秦安大地湾印象等星级宾馆建设,提升改造现有宾馆酒店,尽快实现县区都有四星级以上宾馆。不断完善旅游基础设施,加大旅游营销力度,丰富景区文化活动,旅游人次、综合收入分别增长25%和26%。

(二)加强商贸基础设施建设。加快天水商贸城建设,力争天水会展中心、市文化艺术馆、3号和4号馆年内竣工运营。做好秦州藉口物流园、麦积东柯物流园前期工作,启动建设天水工商联大厦和天水汽车博览城,搬迁天水瀛池蔬菜市场,加快甘谷浙江商贸城二期、清水兴盛物流园、秦安西部小商品城、天水城市蔬菜仓储分拣中心建设和武山洛门蔬菜批发市场迁建进度。实施集贸市场升级改造和农超对接工程,统筹建设县区综合市场、专业市场、特色街区及社区市场。

(三)构建多点支撑消费格局。落实支持服务业政策措施,改造提升餐饮住宿、交通运输等传统服务业,培育研发设计、物流配送、市场营销、融资租赁等生产性服务业,积极发展住房消费、养老家政、旅游休闲、教育文体等生活性服务业,不断完善现代服务业体系。

(四)重视培育电子商务业态。加快建设淘宝网“特色中国·天水馆”,实施阿里巴巴集团“千县万村计划”甘谷、秦安试点工作,创建电子商务示范基地。鼓励企业搭建电子商务平台,促进线上线下互动发展。依托信息化农家店网络平台,加强对乡村干部、农民专业合作经济组织成员的培训,支持有条件的县区建立电子商务发展中心和电商孵化器,壮大电子商务供应商、服务商和网店队伍,推动电子商务向各行业拓展。

五、以示范带动为抓手,加快城乡统筹发展步伐

(一)加快县域经济发展。按照“城乡统筹、产城融合、各具特色”的思路,坚持一产调结构、二产扩总量、三产强基础,进一步优化发展规划和产业布局,重点在扩大经济总量、提升综合实力、加快城镇建设、统筹城乡发展上做好工作,形成产业特色鲜明、竞相差异发展的县域经济新格局。高度重视非公有制经济发展,充分发挥非公有制经济人士、非公有制企业、工商联、各类商会在全市经济建设中的重要作用。

(二)加快推进新型城镇化。全面开展“一县十镇二十村”新型城镇化试点,以天定高速公路、庄天二级公路、310国道天水段沿线三条城乡发展一体化示范长廊为重点,加快城镇水、电、路、气、信息等基础设施建设,完善幼儿园、学校、医院等公共服务设施,优化交通条件,增强城镇综合服务功能和要素聚集能力。落实户籍改革政策,引导农民有序向城镇转移,力争城镇化率达到35.56%。

(三)加快美丽乡村建设。围绕“百村示范、千村整洁、水路房全覆盖”目标,推进新村聚居点建设、旧村落改造和传统村落保护,大力开展“一池两改三化”,建成省级示范村15个、市级示范村25个、整洁村305个。

(四)下大气力整治城乡环境。坚持不懈开展“整治城乡环境、推进三城联创、建设美丽天水”活动,加大水源地及其沿线污染防治力度,下决心逐步关停城区小锅炉,着力做好水、空气、固体废物和噪声等污染防治工作,实施交通疏堵工程,整治占路经营和车辆乱停乱靠,从严查处违反城乡规划许可、建筑施工许可的违法建筑,重视整治违规违法采石采砂行为。

六、以改革开放为动力,激发经济社会发展活力

(一)深入推进各项重点改革。落实“营改增”政策,完善政府预算体系,改进财政预算控制方式,全面实行国库集中支付。建立政府债务管理及风险预警机制,政府债务全部纳入预算管理。规范发展小额贷款公司、融资性担保公司、典当行、拍卖行,打击非法集资,加强对实体经济的金融支持。推动企业建立现代企业制度,完善法人治理结构,推动企业上市融资。深化农村综合改革,有序推开农村土地承包确权登记颁证工作,鼓励农民以土地经营权入股合作社和龙头企业,探索开展土地承包经营权和宅基地抵(质)押贷款试点,进一步拓宽农业保险险种和承保覆盖面。深化事业单位分类改革,全面完成县区政府机构改革,落实公车改革和不动产登记政策,实施文艺院团等经营性文化单位改制,开展水务一体化改革试点,推进甘谷全国农村供销社改革实验区试点工作。

(二)高度重视招商引资工作。围绕特色优势产业和新兴产业,发挥园区平台作用,主动承接产业转移,更加注重择商选资,瞄准央企、科技型企业、民营企业和国内外500强企业,突出引“高”、引“新”、引“强”,扎实做好项目前期工作,落实“四个一”工作机制,为投资商提供优质高效服务,跟进落实签约项目,招商引资到位资金增长20%以上。

(三)继续扩大对内对外开放。深化与丝绸之路经济带、关中—天水经济区城市的交流合作,积极参加区域合作组织和重大节会活动,精心筹办公祭伏羲大典暨第26届伏羲文化旅游节,组织好花牛苹果、秦安蜜桃、秦州大樱桃等特色农产品宣传推介营销活动。采取“一票到底、代码共享”形式,开通天水至深圳、上海等航班。积极申报设立天水海关,加快出口产品基地建设,努力扩大外贸规模,力争进出口总额增长7%。

七、以均等服务为目标,协调发展各项社会事业

(一)切实加大科技创新力度。推进天水创新型城市建设,争取国列、省列科技项目50个,组织实施市列科技项目60个、科技惠民项目20个。发挥科技企业孵化基地作用,培育发展高新技术企业,加快国家先进制造高新技术产业化基地建设。加大知识产权保护力度。注重现有人才的培养,做好高层次和紧缺人才的引进,不断壮大领军型人才队伍。

(二)合力推动教育均衡发展。实施第二期学前教育三年行动计划,扩大学前教育覆盖面。加强义务教育标准化学校建设,落实义务教育经费保障和贫困学生资助政策,调整优化中小学布局,缓解城区中小学大班额问题。积极开展高中特色实验学校和省市级示范学校创建工作,促进普通高中内涵发展。重视加强职业教育,整合职业教育资源,提升市卫校、市农校办学规格和层次。高度重视老年教育,支持老年大学发展。加快市一中麦积校区、市实验小学和市幼儿园新建步伐,启动市三中搬迁。全面实施改善义务教育薄弱学校项目,改扩建中小学校舍30万平方米。加强校园周边环境治理,建设平安文明和谐校园。切实加强教师队伍建设,加大师德师风督查考核,实施教师培训项目,选好配强校长,提高教育教学质量。

(三)大力发展文化体育事业。实施弘扬社会主义核心价值观“人知人晓”、“人信人守”工程,着力抓好“乡村舞台”建设,广泛开展精神文明创建活动。加快市文化中心、市体育中心建设,推进县区文化馆、博物馆、数字图书馆、数字影院建设,做好广播电视数字化改造,加强非物质文化遗产保护传承,打磨提升《大秦文公》、《轩辕大帝》、《关山月》等精品剧目。开展全民健身活动,筹办市第四届中学生运动会,承办全国龙舟邀请赛和三个全国性自行车赛事。

(四)提升卫生计生服务水平。深化医药卫生体制改革,积极推进公立医院综合改革,三级医院逐步取消以药补医和药品加成,二级以上公立医疗机构全面配备并优先使用基本药物,县级以上医疗机构基本药物在省级平台采购。整合卫生计生资源,鼓励支持社会办医,建立健全医师多点执业和分级诊疗制度,推进县(区)乡(镇)村卫生服务一体化管理。加强基层中医药服务体系建设,推动中医药相关产业综合发展。全面落实计划生育政策,稳定低生育水平。实施市中心血站搬迁,加快市妇幼保健院搬迁和秦安、清水中医院业务楼建设,天水全科医师临床培训基地和儿童病区、四〇七医院内科住院楼建成使用。

八、以改善民生为根本,努力增进人民群众福祉

(一)千方百计扩大社会就业。全面落实扶持创业就业各项政策,进一步促进高校毕业生、退役军人、城镇困难人员实现就业,新增城镇就业5.5万人,城镇登记失业率控制在4%以内。多渠道实施城镇创业、农业实用技术、家政服务、农村妇女和“两后生”学历型培训,有效提升创业就业能力,帮助各类困难人员就业。

(二)健全完善社会保障体系。统筹做好社会保险、城乡低保、救灾救济、农村“五保”和城市“三无”老人供养等工作,不断扩大社会保障覆盖范围。适时启动机关事业单位养老保险制度改革,全面推进事业单位和社会组织工伤保险,落实被征地农民养老保险政策,做好村干部养老保险统筹工作,稳步提高城乡居民社会养老保险待遇。积极推进基本医保、大病保险、医疗救助“一站式”即时结报服务。完成麦积全国综合养老示范基地二期工程,建成天水精神病康复医院,加快市残疾人康复中心、县区残疾人托养服务中心和特殊教育学校建设。加强住房公积金管理使用,推进廉租房、公租房并轨运行,年内新开工保障性住房12993套,提供房源4412套,完成13个城中村、31个棚户区、1.2万户农村危旧房改造。

(三)创新提升社会治理能力。以社会治理“66158”工作计划为主线,深化“平安天水”建设,完成“六五”普法规划,推进依法治市进程。完善立体化社会治安防控体系,有效发挥网络监管系统的作用,依法打击各类违法犯罪活动。加强基层基础建设,推进综治管理网格化、信息化,构建政府治理和社会自我调节、居民自治良性互动的格局。创新群众工作方法,做好人民调解和法律援助工作,及时排查化解社会矛盾。开展社会稳定风险评估和舆情分析研判,健全应急处突体系,提高反恐防暴和突发事件处置能力。落实“党政同责、一岗双责、齐抓共管”的安全生产责任制,抓好重点领域和行业安全隐患整治,确保安全生产形势稳定。高度重视民族宗教事务管理,扶持少数民族和民族地区经济社会发展。强化食品药品安全监管,建立食品原产地追溯制度,严惩食品药品违法行为。规范市场秩序,注重价格调节,加强网络监管,整治非法传销,打击非法行医和“医闹”,做好物业服务企业监管工作,切实维护生产者、经营者、消费者合法权益。继续做好关心下一代工作,重视进城务工人员子女就学,关爱农村留守儿童。深入开展“信访法治建设年”活动,做好市委书记、市长留言板反映事项督促办理工作。加强双拥共建、国防后备力量建设,统筹抓好外事侨务、统计调查、公共节能、防震减灾、气象服务、档案史志等工作。

(四)全力以赴办好民生实事。在全面完成省政府改善民生实事的同时,市政府将集中力量办好10件实事。(1)实施“1552”城建民生工程:完成100条小巷道改造,提升改造并免费开放公厕50座,修补人行道5万平方米,建成市区街头小绿地和小花坛200个。(2)完成通行政村沥青或水泥路532条1500公里。(3)落实农村“一事一议”财政奖补项目726个,硬化村组巷道820公里。(4)解决32万农村人口饮水安全问题。(5)完成10个农贸市场提升改造,建成农家店200个,在外地建果蔬直销店20个。(6)启动实施城镇居民大额医疗保险工作。(7)新农合人均筹资标准提高到450元,农村重大疾病新农合保障病种增加到50种。(8)提高农村“五保”和城市“三无”老人供养补助标准。(9)建成村文化室125个,为农村群众放电影3万场、文艺演出50场以上。(10)建成乡镇及社区体育健身中心8个、村级农民健身工程120个。

九、以提高效能为核心,切实加强政府自身建设

(一)全面推进依法行政。把法治思维和法治方式贯穿于工作的全过程,严格按法定权限和法定程序接受监督、行使职权,形成办事依法、遇事找法、解决问题用法、化解矛盾靠法的良好风尚。加强政府法制工作,落实政府法律顾问制度,积极履行行政复议法定职责,不断规范行政执法裁量权。完善政府信息公开制度、决策咨询制度、风险评估制度,按制度办事、靠制度管人、用制度管权,把权力关进制度的笼子,让权力在阳光下运行,努力建设法治政府。

(二)不断提升服务水平。牢固树立宗旨意识,巩固党的群众路线教育实践活动成果,持续整治“四风”问题,始终站在老百姓的角度看问题、想事情、抓工作,着力服务民众、服务企业、服务项目。切实压缩“三公”经费,把有限的资金用于改善基础条件和保障民生,立足打基础、利长远,谋划一批服务民生、发展产业、改善基础设施的大项目,及时破解企业生产经营难题,积极搭建银企对接、企业合作平台,严格整治各种涉企收费行为,加强对建设项目和企业周边环境整治,让其免遭干扰、舒心发展,努力建设服务政府。

(三)加快转变政府职能。进一步推进行政审批制度改革,把该放的彻底放开,该管的切实管好,在继续取消和调整、下放行政审批事项的同时,清理非行政许可审批事项和政府内部管理事项,梳理公开“负面清单”,推进市、县区政府部门“权力清单”、“责任清单”、财政专项资金“管理清单”和政务服务网“三清单一网络”建设,落实减要件、减程序、减环节、压时限的“三减一压”措施,加快电子政务建设,发挥各级政务大厅作用,拓展网上并联审批平台,健全政府督查和绩效考核制度,强化行政效能问责,深化庸、懒、散治理,持续推进作风转变,不断提高政府公信力和执行力,努力建设效能政府。

(四)始终坚持勤政廉洁。坚持“两手抓、两手硬,双促进、双落实”,严格落实党风廉政建设主体责任,严格遵守廉洁从政各项规定,严格执行中央“八项规定”、国务院“约法三章”、省委“双十条”规定和市委“二十七条”意见,加强对重点领域、关键环节、重要岗位权力运行的监督管理,进一步规范政府采购、行政审批、工程招投标、土地出让、探矿采矿、企业改革、财政资金管理等工作,落实农村集体“三资”管理制度,加强审计监督,限期整改审计发现问题,强化行政监察,增强问责刚性,实行“一案双查”,切实加强政府廉政建设,坚决防止和纠正损害群众利益的不正之风,努力建设廉洁政府。

各位代表,改革催人奋进,发展任重道远。让我们倍加珍惜来之不易的发展势头,倍加珍惜深化改革的难得机遇,倍加珍惜团结和谐的良好局面,在市委的坚强领导下,鼓足干劲,砥砺奋进,扎实开展“工作落实年”活动,为建设富裕文明、幸福美好新天水而努力奋斗!

《政府工作报告》名词解释

稳中有升、稳中有进、稳中提质 经济总量有了新提升,经济结构调整有了新进展,经济发展质量有了新提高。

地理标志证明商标 是由国家工商总局依据《中华人民共和国商标法》核准注册,标明某商品来源于某地区,且商品的特定质量或其它特征主要由该地区的自然因素或人文因素决定的标志。

“一县十镇二十村”新型城镇化试点 是《天水市新型城镇化试点工作实施方案》确定的试点单位,“一县”指武山县(也是省列试点);“十镇”指秦州区皂郊镇和天水镇、麦积区甘泉镇和麦积镇、甘谷县磐安镇和六峰镇、秦安县陇城镇和莲花镇、清水县红堡镇、张家川县恭门镇;“二十村”指以上10个镇的20个试点村。

营改增 国家税务总局实施的营业税改为增值税的结构性减税措施,即改变原来对产品征收增值税、对服务征收营业税的方式,将产品和服务一并纳入增值税征收范围,不再征收营业税。

“三城”联创 天水市同步推进国家园林城市、国家卫生城市和中国最佳历史文化旅游城市创建活动。

乡村舞台 是整合农村宣传教育、体育健身、科技推广等资源,利用乡镇文化站、村文化室、农家书屋等阵地,组建村级民间文化社团,建立群众自娱自乐的综合性文化服务中心。

“两横三纵”城市化战略布局 以陆桥通道、沿长江通道为两条横轴,以沿海、京哈京广、包昆通道为三条纵轴,以轴线上主要的城市群地区和城市为支撑,构建我国城市化战略新格局。我市是陆桥通道横轴和包昆通道纵轴上的节点城市。

智慧城市 运用物联网、云计算、大数据、空间地理信息集成等新一代信息技术,促进城市规划、建设、管理和服务智慧化的新理念和新模式。

六大百亿元产业集群 将我市机械制造、电工电器、电子信息、医药食品、建筑建材、能源化工六大主导产业打造成为年销售收入上百亿元的产业集群。

一区多园 “一区”指国家级天水经济技术开发区,“多园”指开发区核心区、辐射区内的各类工业园区和工业集中区。各类工业园区(工业集中区)同挂开发区牌子、同享优惠政策。

“两化”融合 信息化和工业化实现彼此交融。

西北中小企业云平台 采用先进信息技术,依托计算机设备和网络,提供语音通信、数据通信、行业应用等一揽子解决方案,满足中小企业生产、销售、办公和人员管理及电子商务等信息化应用需求的综合信息服务体系。

“48113”现代农业推进计划 我市发展现代农业的战略举措,即做强“果品、蔬菜、畜牧、劳务”4大主导产业,建设蔬菜科技、设施蔬菜、现代农业综合开发、核桃产业、蜜桃产业、循环农业、畜牧产业、花牛苹果产业8大市级现代农业示范园区,实施“10个百万工程”,培育100强龙头企业,打造3条城乡发展一体化示范长廊。

“1461”扶贫攻坚工程 天水市加快新一轮扶贫攻坚的重点工作布局,即重点抓好10个集中连片开发、集中实施40个整乡推进、高质量完成650个整村推进、“雨露计划”培训10万人次。

“136”工程 天水建设华夏文明传承创新区的总体思路,即“一长廊”(天水百里石窟艺术长廊)引领、“三园区”(中华始祖文化园、大地湾国家考古遗址公园、麦积山文化旅游产业园)支撑、“六工程”(文化遗产保护利用工程、历史文化名城保护工程、文化产业发展壮大工程、文化服务基础建设工程、文化旅游融合发展工程、文化品牌打造提升工程)推进。

千县万村计划 阿里巴巴集团在三至五年投资100亿元,建立1000个县级电子商务运营中心和10万个村级电子商务服务站。我市甘谷、秦安两县被列为试点县。

一池两改三化 修建村内垃圾池,改造危房和危墙,硬化、绿化、净化村庄巷道。

“66158”工作计划 天水市社会治安综合治理总体布局,即建立6大体系,实施6大工程,健全10大机制,推进5化建设,实现8大突破。

权力清单、负面清单、责任清单 “权力清单”是给政府划定界限,明确政府应该干什么,“法无授权不可为”。“负面清单”是给企业明确权限,让企业明白不该干什么,可以干什么,“法无禁止皆可为”。“责任清单”是规范政府与市场的关系,明确政府该怎么管市场,“法定职责必须为”。

两手抓、两手硬,双促进、双落实 省委落实党风廉政建设主体责任提出的总体要求,即一手抓改革发展稳定、一手抓党风廉政建设两手都要硬;促进经济社会发展全年目标任务的落实,促进党风廉政建设主体责任的落实。

国务院约法三章 政府性楼堂馆所一律不得新建,财政供养人员只减不增,“三公”经费只减不增。

农村集体“三资” 农村集体资金、集体资产、集体资源。

一案双查 纪检监察机关在查处案件时,既要查清当事人的违纪问题,又要查清主管领导或分管领导的责任范围及责任。
